# Case Report: Drug-induced pneumonia caused by moxifloxacin and a literature review

**DOI:** 10.3389/fmed.2025.1467001

**Published:** 2025-05-07

**Authors:** Zhan Gao, Yunqiu Jiang, Mingzhou Zhang, Chenjing Luo, Zhenghua Wei, Daohui Gong, Guansong Wang

**Affiliations:** Department of Pulmonary and Critical Care Medicine, Xinqiao Hospital, Third Military Medical University (Army Medical University), Chongqing, China

**Keywords:** moxifloxacin, drug-induced pneumonia, allergic reaction, hypersensitivity syndrome, adverse reactions

## Abstract

Drug-induced pneumonia is a rare and potentially life-threatening adverse drug reaction. Moxifloxacin is a fluoroquinolone antibiotic with broad-spectrum antimicrobial activity. Despite reports of moxifloxacin-related side effects such as interstitial nephritis, recurrent tendinitis, and pseudoallergic reactions, moxifloxacin-induced pneumonia is exceedingly rare. We report the case of a 45-year-old male who developed fever and cough, and progressed to hypersensitivity syndrome related to drug-induced pneumonia following moxifloxacin therapy. Discontinuation of moxifloxacin led to resolution of fever with significant resolution of pulmonary lesions. Comprehensive laboratory investigations ruled out other causes, confirming drug-induced pneumonia due to moxifloxacin. This case report provides typical clinical manifestations and pulmonary imaging changes, as well as an analysis of differential diagnosis of pulmonary lesions and key management strategies. The case and related literature review contribute to enhancing our understanding of moxifloxacin-related pneumonia, with important clinical significance in promptly correcting adverse reactions and improving patient outcomes.

## Introduction

Drug-induced pneumonia is an iatrogenic condition caused by drugs and their metabolites, leading to pulmonary inflammation through direct cytotoxicity and allergic reactions (1). It primarily affects the interstitial lung tissue and is termed drug-associated interstitial lung disease (DI-ILD). DI-ILD represents a distinct subtype of interstitial pneumonia. Epidemiological studies report an annual incidence of DI-ILD ranging from 4.1 to 12.4 cases per million population, accounting for 3–5% of all interstitial lung disease (ILD) cases (2). Clinical assessment of drug-induced pneumonia is challenging, as it is typically diagnosed through exclusion. Both the incidence of drug-induced pneumonia and the spectrum of causative drugs have evolved temporally. Before 1980, the main causative drugs were anticancer drugs and gold salts. In recent years, there has been an increase in pneumonia cases caused by antibiotics, chemotherapeutic drugs and anti-inflammatory drugs (3). Notably, 6–26% of DI-ILD cases in contemporary studies are associated with antibiotic use (4–9).

Moxifloxacin is a fluoroquinolone antibiotic widely used for respiratory tract infections (10). It exerts antimicrobial effects by inhibiting DNA gyrase in Gram-negative bacteria and topoisomerase IV in Gram-positive bacteria, resulting in DNA cleavage and rapid bactericidal activity (11). Although fluoroquinolones are generally regarded as having a favorable safety profile, they are associated with a non-negligible incidence of adverse effects. Common adverse reactions to moxifloxacin include QT interval prolongation (12), hepatic and renal dysfunction, mental behavioral abnormalities, gastrointestinal, central nervous system, and skin reactions (13). Rare side effects such as recurrent tendonitis (14), interstitial nephritis (15), and allergic-like reactions, bilateral acute iridocyclitis (16), and cardiotoxicity have also been reported. Despite these known risks, moxifloxacin-induced pneumonia remains scarcely reported. To our knowledge, there are literature reports of pulmonary involvement in DRESS syndrome caused by moxifloxacin, which is considered to be related to allergic reactions.

Herein, we present a rare case of moxifloxacin-triggered pneumonia in a 45-year-old male, alongside a discussion of diagnostic strategies and a review of severe adverse reactions attributed to this agent. Written informed consent was obtained from the patient for publication.

## Case presentation

**Figure 1:**
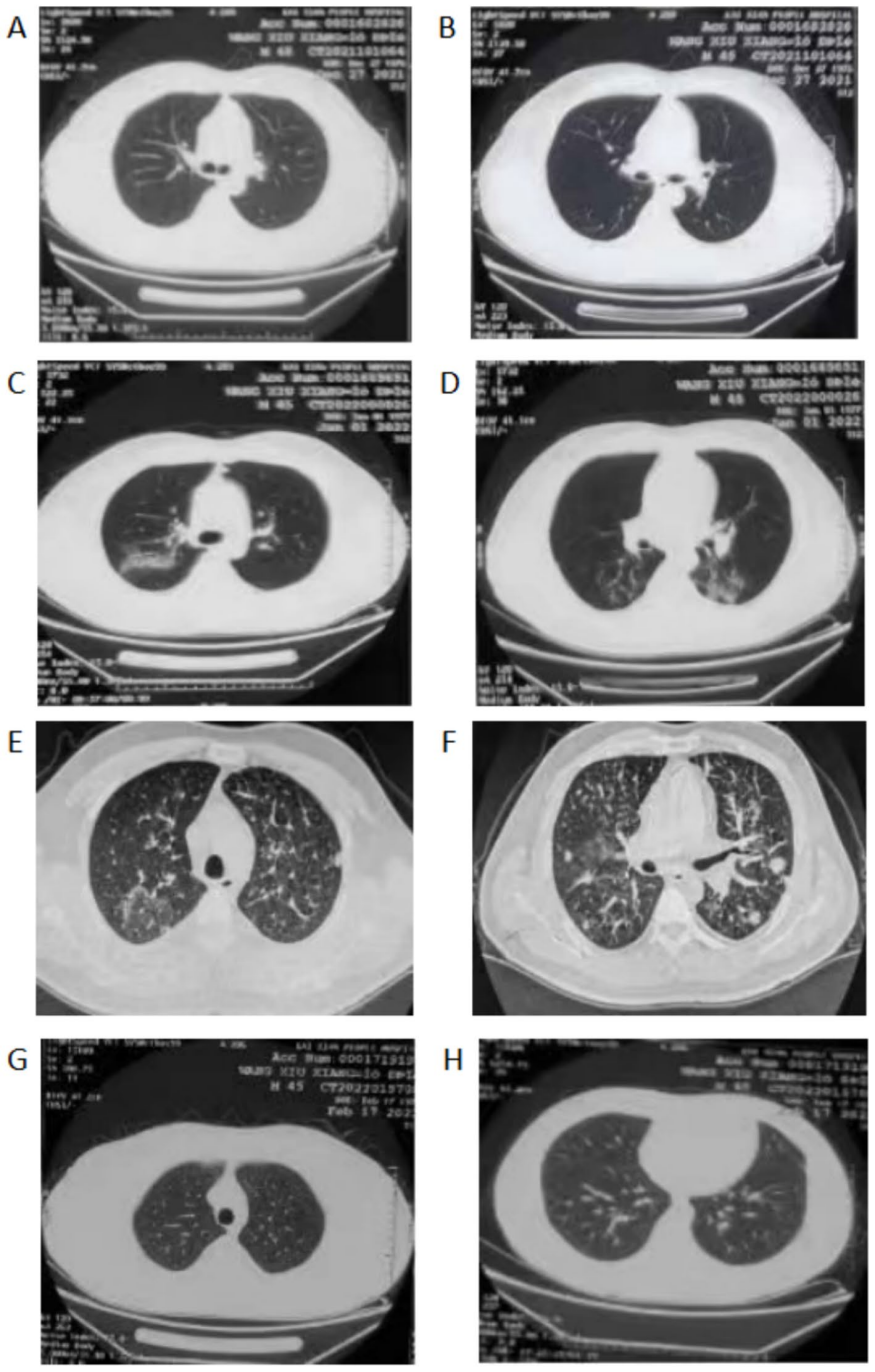
CT images of patients at different times [**(A,B)** show the lung CT of D-10, **(C,D)** show the lung CT of D-5, **(E,F)** show the lung CT of D 9, **(G,H)** show the lung CT of D 47].

**Figure 2:**
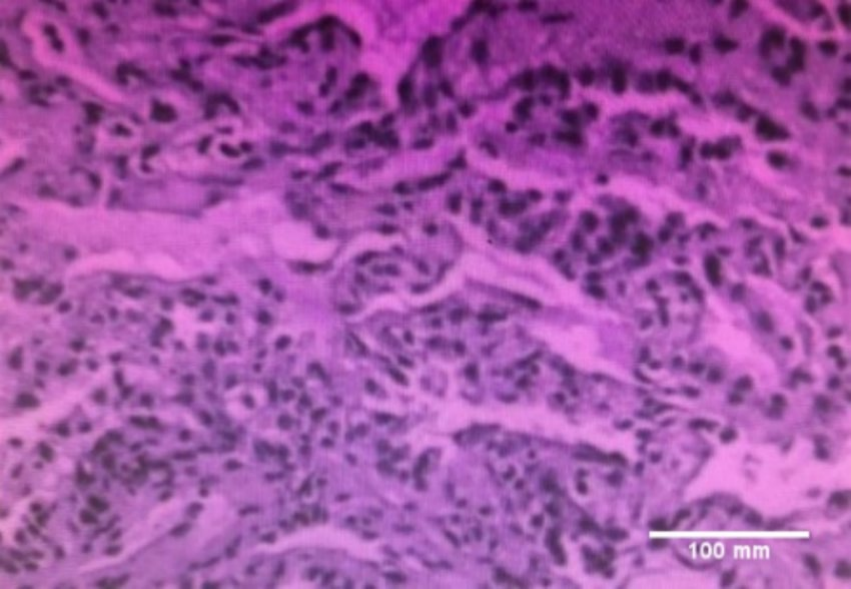
Pathology of the posterior segment of the right upper lobe lung. Bronchopulmonary biopsy was performed on the posterior segment of the upper right lobe, and pathology revealed chronic inflammation with fibrous tissue hyperplasia. Scar bar = 100 mm.

A 45-year-old male patient was admitted with a persistent cough of over 10 days. He had no history of hypertension, diabetes, smoking, or alcohol consumption. The patient experienced paroxysmal coughing for over 10 days after catching a cold, with exacerbated symptoms at night, producing a small amount of sticky white sputum that was difficult to expectorate. He did not present with chills, high fever, purulent sputum, night sweats, chest pain, hemoptysis, or respiratory distress. Hospitalization at the local hospital revealed mild lung infection on chest CT (Figures 1A,B) and no significant abnormalities in the complete blood count and renal and liver function tests. He was then treated with moxifloxacin 400 mg IV daily, however, five days later, he developed fever, with a maximum body temperature of 39.6°C, without apparent chills or shivering. Moxifloxacin was discontinued, and the antibiotic was changed to piperacillin-tazobactam sodium 4.5 g IV q8h. Post-antibiotic treatment, a follow-up chest CT revealed infective lesions in the right upper lobe and bilateral lower lobes that were significantly more extensive than before (Figures 1C,D). The patient came to our hospital for further treatment, upon admission, laboratory investigations showed a C-reactive protein (CRP) of 82.9 mg/L and WBC count of 4.5 × 10^9^/L, with a neutrophilic count of 2.4 × 10^9^/L. He was once again administered moxifloxacin 400 mg IV daily for the infection and underwent a bronchoscopy examination 3 days later, yielding no significant findings. Bronchopulmonary biopsy was performed on the posterior segment of the upper right lobe, and pathology revealed chronic inflammation with fibrous tissue hyperplasia (Figure 2). Both the Periodic Acid-Schiff staining and Cytomegalovirus (CMV) tests were negative. During this period, his cough and sputum improved, and his CRP reduced to 6.8 mg/L. However, on the 6th day of admission, the patient developed a fever with a body temperature of 38°C, without chills, shivering, runny nose, sneezing or other symptoms. The lavage Metagenomic next-generation sequencing did not reveal significant bacteria, viruses, or mycobacterium tuberculosis. Symptomatic treatment was administered, and a repeat CRP measurement recorded 114.2 mg/L. The blood routine test showed a WBC count of 4.57 × 10^9^/L, with a neutrophil count of 3.48 × 10^9^/L and a normal eosinophil count (0.3×10^9^/L), with no observed rash. The patient was given piperacillin-tazobactam sodium 4.5 g IV q8h for the infection; however, the fever persisted, and his cough and sputum worsened, with shortness of breath during fever. Acyclovir (0.25 g IV daily) antiviral treatment was added for 3 days, and a follow-up chest CT on the 9th day of hospitalization revealed scattered nodular lesions, ground-glass opacity, and interlobular septal thickening in both lungs (Figures 1E,F). The results of the antinuclear antibody spectrum and anti-neutrophil antibody testing were negative. Moxifloxacin was discontinued on the 10th day of hospitalization, after which the patient remained afebrile and his cough improved. Two days later (January 17, 2022), methylprednisolone 40 mg daily was added for anti-inflammatory treatment, and a follow-up chest CT on the 17th day of hospitalization showed significant resolution and improvement of the lung lesions. Upon discharge, the patient continued to take oral Prednisone 30 mg daily, with a weekly reduction of 5 mg. A follow-up chest CT 1 month later showed complete resolution of the lesions (Figures 1G,H), with no significant coughing or sputum symptoms. During the hospitalization period, the patient took compound licorice oral liquid for cough relief (the patient had used it previously without adverse reactions), with no other special medications.

**Figure 3:**
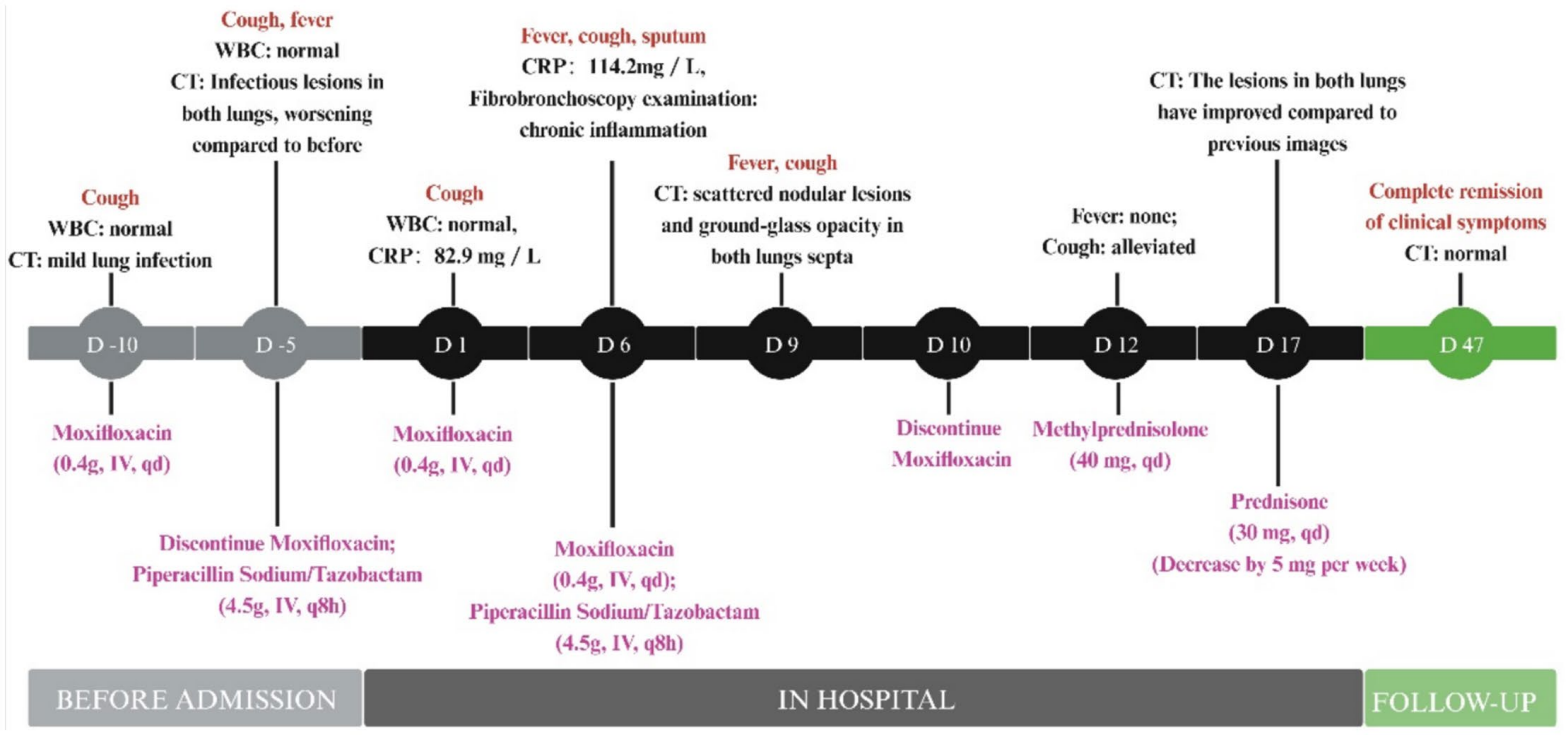
Flow chart of patient diagnosis and treatment.

In summary, the patient exhibited symptoms of fever and pneumonia following two courses of moxifloxacin therapy. The temporal association and resolution after discontinuation supported the diagnosis of moxifloxacin-induced fever and pulmonary changes. Throughout the treatment process, the patient actively communicated his symptoms and concerns, enabling timely adjustments to the care plan and ultimately achieving favorable outcomes. The diagnostic and therapeutic timeline is summarized in Figure 3.

### A literature review of adverse reactions caused by moxifloxacin

Moxifloxacin, a broad-spectrum antibiotic with favorable tissue penetration and a prolonged half-life, is widely used in the clinical management of infectious diseases. Notably, moxifloxacin may modulate pulmonary vascular permeability (17). A study reported that moxifloxacin administration and CMV replication within the first year post-transplantation were associated with an elevated risk of skin squamous cell carcinoma (SCC) (18). Specifically, moxifloxacin exposure increased the risk for SCC development during follow-up [hazard ratio (HR) = 2.9, 95% CI: 1.5–5.7; *p* = 0.001].

Drug-induced hypersensitivity syndrome (DIHS), also known as drug reaction with eosinophilia and systemic symptoms (DRESS), is a severe adverse reaction syndrome caused by moxifloxacin, characterized by systemic involvement includes pulmonary manifestations. Zhang et al. (19) reported a case of a 47-year-old woman who developed cough, fever, rash, hematologic abnormalities, shortness of breath, and interstitial lung changes after oral moxifloxacin therapy. Another report documented a patient with an upper respiratory infection who developed moxifloxacin-induced hypersensitivity pneumonitis. High-resolution chest CT scan revealed diffuse interlobular septal thickening and ground-glass opacities in both lower lung lobes. Skin prick testing and enzyme-linked immunosorbent assay for detecting specific IgE antibodies to moxifloxacin yielded negative results (20).

The hypersensitivity response caused by moxifloxacin also involves other organs, Chatzikyrkou et al. (15) reported a case of moxifloxacin-induced acute interstitial nephritis, renal biopsy revealed dense eosinophilic infiltrates and severe interstitial edema. Other severe adverse reactions, such as acute generalized exothermic hypersensitivity (21), Stevens Johnson syndrome (22), toxic episode neolysis and acute life failure (23), all of which are linked to the hypersensitivity reaction of moxifloxacin (Table 1). Fortunately, prompt recognition and accurate diagnosis can significantly improve clinical outcomes.

**Table 1:** Summary of adverse reactions caused by moxifloxacin.

	Adverse reactions					
Features	DRESS	AKI	ALF	SJS	TEN	AGEP
Latentperiod	2–6 weeks	2–3 weeks	1–4 weeks	36 h	1 weeks	3–6 weeks
Fever	Yes	Yes	No	Yes	No	No
Skin damage	Yes	No	No	Server	Server	Yes
Viscus damage	Yes	Yes	Yes	Yes	Yes	Yes
Pathology	Mild keratinization of the epidermis, swelling around small blood vessels in the dermis, and proliferation of collagen fibers	Interstitial edema with eosinophilic infiltration	None	None	None	None
Treatment	None	Drug withdrawal + hormone therapy	Drug withdrawal + *combined therapy*	Drug withdrawal	Drug withdrawal	drug withdrawal

DRESS, drug reaction with eosinophilia and systemic symptoms; AKI, acute kidney injury; ALF, acute liver failure; SJS, stevens-johnson syndrome; TEN, toxic epidermal necrolysis; AGEP, acute generalized exanthematous pustulosis,

## Discussion

Antibiotics remain the cornerstone of treating respiratory infectious diseases. In clinical practice, attention must be paid not only to antibiotic allergies and resistance, but also to potential adverse drug reactions (24). Although moxifloxacin-associated adverse effects are widely reported, cases of moxifloxacin-induced pneumonia remain scarce. Diagnosing moxifloxacin-induced pneumonia is particularly challenging, as non-infectious lung injury caused by moxifloxacin often mimics bacterial or viral pneumonia and requires exclusion of infectious etiologies to avoid life-threatening delays in management.

In the currently reported cases, hypersensitivity symptoms (fever, pulmonary inflammation) typically emerged 5 days after initiating moxifloxacin. Discontinuation led to prompt defervescence and radiographic improvement on chest CT. Notably, moxifloxacin-induced pneumonia lacks pathognomonic features. CT findings consistently demonstrate pulmonary infiltrates (20), and diagnosis relies on temporal associations between drug exposure/symptom resolution. The Naranjo Adverse Drug Reaction Probability Scale aids causality assessment, particularly when reactions are reversible or reproducible upon rechallenge. In this case, re-exposure to moxifloxacin triggered fever and radiographic progression, which resolved after withdrawal (Naranjo score = 6, “probable” causality) (25), fulfilling diagnostic criteria for drug-induced pneumonia (26).

Mechanistically, drug-induced pneumonia primarily manifests as interstitial lung disease through dual pathways: 1. Direct cytotoxicity: Moxifloxacin metabolites may damage alveolar epithelial and capillary endothelial cells, disrupting lung architecture and inciting inflammation (27). 2. Immune-mediated injury: Moxifloxacin metabolites act as haptens, forming antigenic complexes that trigger T-cell-dominated immune responses. This involves cytokine release, T-cell activation (3, 27), and delayed-type hypersensitivity (evidenced by normal IgE levels and symptom latency) (28). Cross-reactivity among quinolones is common due to MHC-dependent T-cell recognition (21, 29, 30). Notably, rapid symptom resolution post-drug withdrawal aligns with acute hypersensitivity-driven injury, akin to nitrofurantoin-induced pneumonitis (31). However, whether moxifloxacin’s immunomodulatory properties directly contribute to lung injury remains unclear, warranting further mechanistic exploration.

This study has several limitations. First, the classification of moxifloxacin-associated pneumonia as interstitial pneumonia in this case was not definitively confirmed through histopathological examination. Second, the precise pathophysiological mechanisms underlying moxifloxacin-induced lung injury remain poorly characterized, necessitating further mechanistic investigations. Additionally, we were unable to obtain patient perspective due to privacy constraints.

In conclusion, this case underscores moxifloxacin as a potential culprit in drug-induced pneumonia and highlights the imperative to consider pharmacologic etiologies in patients with unexplained fever and pulmonary infiltrates.

## Data Availability

The original contributions presented in the study are included in the article/supplementary material, further inquiries can be directed to the corresponding authors.
